# Exit examination: a survey of UK psychiatrists' views

**DOI:** 10.1192/pb.bp.114.046805

**Published:** 2015-10

**Authors:** Nicholas S. Hughes, Angela Haselgrove, Matthew S. Tovey, Waqqas A. Khokhar, Muj Husain, Victoria C. Osman-Hicks

**Affiliations:** 1 Rohallion Clinic, NHS Tayside; 2 Royal Edinburgh Hospital, NHS Lothian; 3 Reaside Clinic, Birmingham and Solihull Mental Health NHS Foundation Trust; 4 Leicestershire Partnership NHS Trust; 5 Academy of Medical Royal Colleges' Trainee Doctors' Committee (ATDG); 6 Southern Health NHS Foundation Trust

## Abstract

**Aims and method** The Royal College of Psychiatrists is considering how best to introduce a post-MRCPsych-examination assessment (‘exit examination’) in anticipation of external pressures to ensure patient safety through the use of such assessments. The Psychiatric Trainees' Committee conducted an online survey to gather the views of psychiatrists regarding the possible format and content of this examination in the hope that this information can be used to design a satisfactory assessment.

**Results** Of the 2082 individuals who started the survey, 1735 completed all sections (83.3%). Participants included consultants and trainees from a range of subspecialties. There was general agreement that the content and structure of the exit examination should include assessment of clinical and communication skills.

**Clinical implications** UK psychiatrists believe that an exit assessment should focus on clinical and communication skills. It should assess both generic and subspecialty-specific competencies and incorporate a mixture of assessment techniques.

The General Medical Council (GMC) is currently undertaking a review of the training and assessment of doctors in the UK, with a view to making a number of recommendations to improve the quality of training.^1^ One of the main aims of the GMC in the area of training and education, as defined in their education strategy for 2011–2013, is that:

‘To ensure consistency and clarity, we will define clear outcomes which must be met by students and trainees on the completion of different stages of training.’^1^

Although higher specialist training examinations are not explicitly mentioned, the introduction of a clear means of ensuring that all doctors have been assessed as competent to practise independently before they are granted a Certificate of Completion of Training (CCT) is an important aspect of the GMC document. There is also inference that a final annual review of competence progression alone is not sufficiently rigorous and that some form of final, standardised assessment (an exit examination) is necessary. It has been acknowledged that an exit examination must be relevant and knowledge based, but its form and content has been left for individual medical Royal Colleges to decide.

Since 2008, psychiatric trainees have been required to sit three written papers and undertake one clinical examination (Clinical Assessment of Skills and Competencies, CASC) before gaining Membership of the Royal College of Psychiatrists (MRCPsych).^2^ Before 2008, trainees would sit two written papers, an Objective Structured Clinical Examination (OSCE) and a long case examination. This increase in written papers was also accompanied by raising examination fees. These changes were viewed negatively by many trainees as it placed them under greater time and financial pressures.^3^ Further disquiet was caused among trainees in 2011, when it was revealed that the College had made a financial surplus from examination fees.^4^ In response to trainee concerns, the College has reduced the number of written papers to two and also cut exam fees.

In this historical context, there are fears that the introduction of an exit examination might be perceived unfavourably. With this in mind, the Psychiatric Trainees' Committee (PTC), an elected group of psychiatric trainees from across the UK supported by the Royal College of Psychiatrists, established an Examinations Working Group in 2012, which set the following priorities:

- to ensure that the exit examination assesses what is really important for new consultants to ensure safe and effective care of patients
- to ensure the views of trainees have a major role in informing what the examination will look like and what it will assess.

Gaining the views of current trainees and consultants was seen as the necessary first step in working towards these priorities.

## Method

### Questionnaire development

A questionnaire was created by members of the PTC Examinations Working Group (see online supplement DS1). The first draft was piloted by an opportunistic sample of PTC members to determine where modifications were needed. Advice was obtained from senior officers within the Royal College of Psychiatrists and further modifications were made. In May 2013, the survey was uploaded to www.surveymonkey.com, an online survey hosting website.

A range of free-text responses were available. Owing to the unexpectedly large volume of such comments received, it was retrospectively agreed that they would be reported separately.

### Recruitment of participants

An email was sent to all Members and Pre-Membership Psychiatric Trainees included in a mailing list held by the College (see online supplement DS2). In addition, PTC members were encouraged to pass on details of the survey to trainees and consultants locally. The survey commenced on 22 May 2013; responses were accepted up until 29 July 2013 (the initial deadline of 21 June was extended because the rate of responses remained high). All those who opted in to completing the survey were included in the final analysis.

### Analysis

Data were separated with respect to participant seniority and by subspecialty. Subspecialty groups comprised both higher trainees and consultants. Given the categorical nature of the data collected, chi-squared tests were applied to identify differences between groups; calculations were performed on GraphPad, an online data analysis tool (http://graphpad.com/quickcalcs). It was anticipated that not all those who commenced the survey would complete all sections due to time constraints or distractions. It was agreed beforehand that all who had responded to any given question would be included in the analysis of that part of the survey.

For sample data to be accurate, they need to be representative of the population under consideration. Unfortunately, we were not able to ensure this because of governance difficulties. The mathematical theorems which justify standard statistical procedures apply only to random samples and so our statistical findings cannot be accepted as exact.

## Results

### Grade and specialty of survey respondents

Overall, 10 298 consultants and trainees were sent an email inviting them to take part in the survey. No email address was available for an additional 371 (3.6%) consultants and trainees in the College database. About a fifth of those contacted (*n* = 2082) started the survey and 1735 completed all sections (83.3%). These respondents included 487 core trainees (23.4%), 509 higher trainees (24.5%), 297 consultants with less than 5 years' experience (14.3%) and 788 consultants with more than 5 years' experience (37.9%).

Among higher trainees and consultants, there was a range of responses across the psychiatric specialties: 222 child and adolescent (10.7%), 146 forensic (7.0%), 810 general adult (38.9%), 117 intellectual disability (5.6%), 275 old age (13.2%) and 53 psychotherapy (2.5%) specialists.

### Preferred content of assessment

**Fig 1:**
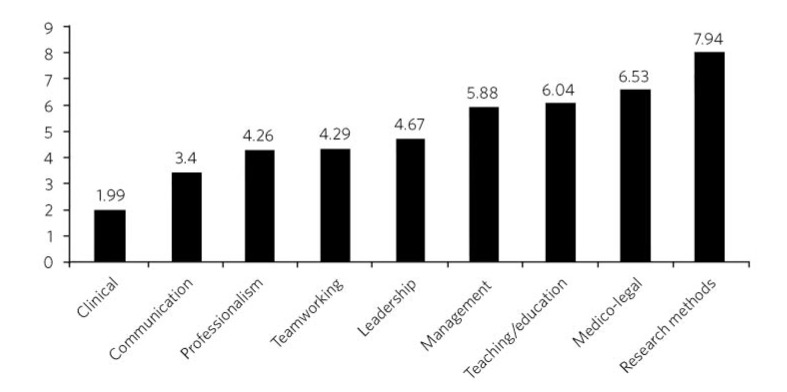
Mean ranking score of exam content components across all survey participants (lower score indicates stronger preference); *n* = 1896.

In general, respondents considered clinical and communication skills to be the most important items to be assessed in an exit examination (*n* = 1896; Fig. 1); research methods, medico-legal issues, teaching and education and management were considered of lesser importance.

### Examination content preferences by grade

There was little variability in the overall ranking of examination content when the data were separated with respect to seniority of survey participant. Clinical and communication skills were ranked first and second across all groups. Professionalism, team-working skills and leadership skills accounted for the next three components across all grades, although there were minor variations in their order, with senior consultants uniquely rating team-working skills above professionalism. In all groups, management skills, teaching skills and medico-legal issues were the next three components. Senior consultants considered teaching skills to be more important than the other two components, but consultants with less than 5 years' experience considered teaching skills less important, with a greater emphasis on management and medico-legal skills. In all groups, research skills were considered to be the least important component of any proposed exit examination.

**Fig 2:**
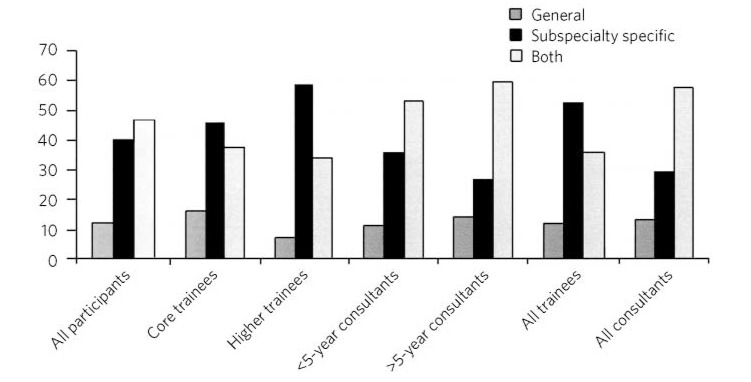
Preferences about subspecialty specificity by grade (%)

Consultants and trainees differed in their views regarding whether the exit examination should be specialty specific, general or a mixture of the two (*P*<0.001; Fig. 2). The majority of trainees (*n* = 472; 52.3%), including 58.4% of higher trainees, thought that an exit examination should be unique to each psychiatric subspecialty, whereas consultants were predominantly of the opinion that it should comprise both subspecialty and general components (57.3% of all consultant participants).

### Examination content preferences by subspecialty

Despite the fact that the general pattern of rankings of examination content was similar to the overall ratings across subspecialties, there were some notable differences between specialty groups.

Clinical and communication skills were again ranked first and second in terms of importance for inclusion in an exit examination across all specialties. As was the case when the item rankings were separated by grade, professionalism, team-working and leadership were ranked in positions 3–5 across all specialties, although team-working skills were considered particularly important by those from the general adult, old age and medical psychotherapy Faculties.

Management and teaching skills were the items considered next by all groups except forensic psychiatrists. Forensic psychiatrists rated medico-legal skills higher than all other subspecialties at 6th *v*. 8th by all others. There was again a consistent view that research skills were the least important item to assess as part of an exit examination.

All subspecialties were consistent in favouring a mixture of subspecialty and general components to any proposed exit examination, with the exception of child and adolescent psychiatry, where 63.6% of respondents favoured a subspecialty-specific exit examination (*P*<0.001).

### Structure preferences

Across the whole sample (*n* = 1818) the majority of respondents (*n* = 922, 50.7%) were in favour of a mixture of practical, written and oral components; 361 (19.9%) favoured an oral examination alone, 285 (15.7%) opted for a practical examination and for 250 (13.8%) a written examination was the preferred option.

**Fig 3:**
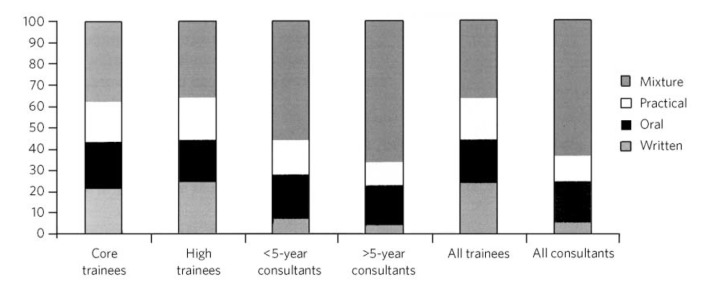
Percentage of respondents preferring each type of exit examination structure by grade.

There were no significant differences between core and higher trainees in the overall distribution of responses given (*P* = 0.65). A mixture of practical, written and oral examination components was the preferred option across all groups regardless of grade, but significantly more popular with consultants (with a clear majority in favour) than trainees (*P*<0.0001). On the other hand, a purely written examination was significantly more popular with trainees than with consultants (*P*<0.001; Fig. 3).

A mixture of all three examination components was favoured by participants from all subspecialties. More than half of all participants preferred this option in all subspecialties except for intellectual disability psychiatry and there was a significant difference in the exam structure preferred by intellectual disability psychiatrists compared with the other subspecialties (*P* = 0.003). No significant differences were noted between the preferred exit examination structure of the other disciplines (*P* = 0.25).

#### Preferences for a written examination

We received 1818 responses about preferences regarding the format of the written component of any proposed exit examination. The most popular option overall was a reflective report about a clinical scenario and associated *viva* (37.2%). Short-answer questions and multiple choice questions (MCQs) were less popular, representing 23.3% and 23.2% of responses respectively. The least popular options were extended matching questions (EMQs; 10.1%) and essay writing (6.2%).

There was evidence of a clear difference in the preferred format of a written examination between trainees and consultants (*P*<0.0001). Overall, trainees preferred MCQs (36.2%); however, core trainees were significantly more likely to favour MCQs than higher trainees (*P* = 0.001). The opposite was true with EMQs, with higher trainees being significantly more in favour of their use than core trainees (*P* = 0.009). Among consultants, only 11.5% favoured the use of MCQs; reflective report accompanied by a *viva* was the most popular option for the written component of the exit examination (44%). Essays were the least popular form of assessment by those of all grades, although consultants were nevertheless significantly more in favour of their use than trainees (*P* = 0.007).

There was a significant difference in views about how written examination components should be structured across the subspecialties (*P* = 0.001). Significantly more psychotherapists (*n* = 31, 70.5%) preferred the use of reflective writing and an associated *viva* than the other subspecialties (*P*<0.0001). Excluding psychotherapy, there were no significant differences between subspecialties regarding their views about the use of MCQs (*P* = 0.98), EMQs (*P* = 0.1), brief assessment questions (*P* = 0.12) and essay writing (*P* = 0.21). There was evidence of a significant difference with regard to views about reflective practice (*P* = 0.03): this was popular among intellectual disability psychiatrists (48.1%) yet less favoured by forensic psychiatrists (30.1%).

#### Preferences for a practical examination

For two-thirds of respondents (*n* = 1197, 65.8%) assessments in the workplace (workplace-based assessments, WPBAs) were the preferred option for a practical examination; 621 (34.2%) were more in favour of the OSCE format. Higher and core trainees expressed a strong preference for the use of WPBAs over OSCEs, with a strong preference in both groups (80% of higher trainees (*n* = 353) and 80.4% of core trainees (*n* = 336)). Consultant psychiatrists favoured WPBAs over OSCEs and no significant difference between less experienced and more experienced consultants was found (*n* = 147, 58.1% *v. n* = 361, 51.1%; *P* = 0.067). On the other hand, there was greater support among trainee psychiatrists for WPBAs than among consultants (*n* = 689, 80.2% *v. n* = 508, 53.0%); this was a highly significant difference (*P*<0.0001).

#### Preferences for an oral examination

Across all participants in the survey (*n* = 1818), 677 (37.2%) considered a structured *viva* to be the best option for an oral examination; 434 (23.9%) chose patient management problems (PMPs) and 707 (38.9%) opted for a combination of the two. There was no significant difference between the views of core and higher trainees (*P* = 0.38), who overall favoured the use of a structured *viva* alone (334 of 859 responses, 38.9%). Among consultants, the most popular type of oral examination was a combination of both structured *viva* and PMPs (427 of 959 responses, 44.5%), with no difference between consultants with more than 5 or less than 5 years' experience (*P* = 0.79). There was, however, a significant difference in the consultants' and trainees' preferences regarding any proposed oral exit examination components (*P*<0.0001).

A mixture of PMPs and structured *viva* was the most popular oral examination structure for specialists in child and adolescent psychiatry (43.1%, *n* = 197), forensic psychiatry (45.9%, *n* = 133), general adult psychiatry (40.8%, *n* = 701) and intellectual disability psychiatry (42.6%, *n* = 108). Specialists in old age psychiatry and psychotherapy both preferred a structured *viva* alone (43.6%, *n* = 243 and 36.4%, *n* = 44). The differences between specialty groups did not reach statistical significance (*P* = 0.39).

### A question of awarding a diploma or certificate

Across the whole sample, there was a small majority in favour of awarding a certificate or diploma for any proposed exit examination (50.2%, *n* = 1818); 10.3% were against and 39.5% were unsure or considered this matter unimportant. The numbers decreased with seniority, with 61% (*n* = 418) of core trainees, 56.5% (*n* = 418) of higher trainees, 45.5% (*n* = 253) of junior consultants and 41.5% (*n* = 706) of senior consultants considering a diploma to be necessary following successful completion of the proposed exit examination. The views of trainees and consultants were significantly different (*P*<0.0001).

## Discussion

The prospect of an exit examination to be taken by all psychiatric trainees before they are deemed eligible for a CCT is not new. Even before most current psychiatric trainees were born, Kendell^5^ wrote of his disapproval regarding the possible introduction of an exit examination at a time of major changes in the structure of postgraduate medical education in the UK. Kendell identified potential problems, including likely trainee dissatisfaction and the implications for recruitment into psychiatry. He expressed particular concern about the possible outcomes for those trainees who were unsuccessful in such exit examinations.

In the early 1990s, after the publication of the Calman report,^6^ both the then president of the Royal College of Psychiatrists^7^ and the Collegiate Trainees Committee^8^ (the predecessor to the PTC) spoke out strongly against the possibility of introduction of an exit examination.

Ten years ago, Tyrer & Oyebode^9^ discussed the need for changes to the College membership examinations. They acknowledged that political and other external factors would continue to have an influence on how doctors training to be psychiatrists would be assessed, predicting the likelihood of an exit examination being introduced at some point in the future. Around that time, major changes to the role and function of the GMC were proposed following the publication of the 5th report of the Shipman Inquiry^10^ and an associated growing public interest in the training and monitoring of doctors in the UK. In the intervening decade, there have been a number of reports highlighting concerns about patient safety and quality of care provided under the auspices of the National Health Service.^11,12^

This paper presents one of the first psychiatry trainee- and consultant-wide surveys into exit examination of UK psychiatrists. The survey had a very large number of responses, giving insight into the views of about 2000 consultants and trainees from across the country. This no doubt reflects the understandable anxiety raised by the prospect of an exit examination. Owing to the number of responses we received, free-text comments were not included within this paper, nevertheless they are likely to provide an invaluable range of views that will further assist the College in ensuring that any future exit examination reflects the views of the College members and pre-membership trainees.

It is perhaps surprising that clinical and communication skills were considered the most important factors to be assessed, given that previous studies have demonstrated that these are the areas in which most new consultants feel relatively confident; resource management and supervision have been shown as areas in which new consultants feel underprepared by their training and might therefore be considered more important to assess towards the end of training.^13^ This may reflect the fact that trainees consider an ‘examination’ to be a concrete test of clinical or communication skill or knowledge and may not have considered other assessments, such as reflective writing, to be an ‘examination’. An example of such an alternative assessment is the piloted Wessex advanced training professionalism programme.^14^

The degree to which an exit assessment should be generic for all trainees or should concentrate on testing subspecialty-specific knowledge varied significantly depending on the participant's status. Trainees were significantly more in favour of subspecialty-specific examinations, whereas consultants, particularly those with more experience, favoured a greater mixture of general and specialty-specific assessments. This may reflect the fact that on completion of the MRCPsych examinations, trainees feel confident with general psychiatry and that they consider a detailed knowledge of their subspecialty to be the primary goal of higher training. Those with more experience may value maintaining a broader skills base across the psychiatric disciplines. However, the recent publication of the *Shape of Training* review^15^ and its suggestion of broad-based training and post-Certificate of Specialty Training credentialing may complicate the issue of both when this assessment should take place in training and whether or not there is value in it being general across all psychiatric subspecialties.

Overall, the participants leaned towards a mixture of several different assessment styles for an exit examination. This finding could be explained by a perception that multi-modal assessment techniques increase the fairness, reliability and validity of an examination. Concerns have been expressed in the past by both trainees and consultants that changes made to psychiatric examinations (such as the introduction of CASC in 2008) did little to improve the validity and reliability of clinical examination.^16^

Exploration of views about the awarding of a diploma or similar certificate following successful completion of the exit examination revealed differences between trainees and consultants. A significant majority of trainees thought that such a reward should be provided, yet consultants differed markedly in their view. Given the potential difficulties in marketing the introduction of an exit examination to trainees, this difference in opinion might be something that the College should consider carefully.

### Limitations

Despite the many strengths of this study, it is important to note that in pursuit of a wide range of responses, we were obliged to accept a number of methodological weaknesses that should be considered when interpreting the results. A study of this type is difficult to undertake in such a way as to encourage responses from a broad and representative audience; one of our principal goals was to gain the views from as many relevant individuals as possible. Given the subject investigated, it was essential to allow anonymous responses to the survey and this further limited our ability to control the recruitment of participants. Any sampling technique that would ensure a more demonstrably representative selection of views would have been associated with markedly fewer participants and might have led to the study being unfeasible, because of the difficulties in negotiating the relevant information governance arrangements of the Royal College of Psychiatrists. On balance, we agreed that the best way to obtain as representative a sample as possible in an acceptably efficient fashion would be to accept all responses from an open survey sent to all consultant psychiatrists and trainees known to the College. Consultants comprised 70% of those who were invited to participate, but only 52% of those who participated in the survey. It is perhaps unsurprising that this study would be of greater interest to those more likely to be directly affected by the introduction of an exit examination, but the possibility of bias should be borne in mind when considering results relating to the sample as a whole. We anticipated that the concerns about randomisation were likely to be magnified with regard to the data provided regarding the subspecialties. However, after comparing the survey data with a breakdown of the workforce as detailed in the most recently published census of the College membership,^17^ the distribution of survey respondents and the census data were broadly similar with respect to subspecialty, although the relatively small number of responses from psychotherapy and intellectual disability consultants might make their comments less representative.

In summary, this survey provides an interesting insight into the views of a wide range of trainee and consultant psychiatrists on the nature of any future exit examination. It suggests that overall trainee and consultant psychiatrists consider that if introduced, an exit examination should primarily focus on clinical and communication skills, should assess both generic and subspecialty-specific skills, and should be undertaken using a mixture of different assessment techniques.
